# Role of cystathionine beta synthase in lipid metabolism in ovarian cancer

**DOI:** 10.18632/oncotarget.5424

**Published:** 2015-10-06

**Authors:** Prabir K. Chakraborty, Xunhao Xiong, Soumyajit Banerjee Mustafi, Sounik Saha, Danny Dhanasekaran, Nawajes A. Mandal, Scott McMeekin, Resham Bhattacharya, Priyabrata Mukherjee

**Affiliations:** ^1^ Department of Pathology, The University of Oklahoma Health Sciences Center, Oklahoma City, Oklahoma, USA; ^2^ Department of Obstetrics and Gynecology, The University of Oklahoma Health Sciences Center, Oklahoma City, Oklahoma, USA; ^3^ Department of Cell Biology, The University of Oklahoma Health Sciences Center, Oklahoma City, Oklahoma, USA; ^4^ Dean A. McGee Eye Institute, The University of Oklahoma Health Sciences Center, Oklahoma City, Oklahoma, USA; ^5^ Peggy and Charles Stephenson Cancer Center, The University of Oklahoma Health Sciences Center, Oklahoma City, Oklahoma, USA

**Keywords:** CBS, lipid metabolism, SREBP, ovarian cancer

## Abstract

Elevated lipid metabolism is implicated in poor survival in ovarian cancer (OC) and other cancers; however, current lipogenesis-targeting strategies lack cancer cell specificity. Here, we identify a novel role of cystathionine beta-synthase (CBS), a sulphur amino acid metabolizing enzyme highly expressed in several ovarian cancer cell lines, in driving deregulated lipid metabolism in OC. We examined the role of CBS in regulation of triglycerides, cholesterol and lipogenic enzymes via the lipogenic transcription factors SREBP1 and SREBP2. CBS silencing attenuated the expression of number of key enzymes involved in lipid synthesis (FASN and ACC1). Additionally CBS abrogates lipid uptake in OC cells. Gene silencing of CBS or SREBPs abrogated cellular migration and invasion in OC, while ectopic expression of SREBPs can rescue phenotypic effects of CBS silencing by restoring cell migration and invasion. Mechanistically, CBS represses SREBP1 and SREBP2 at the transcription levels by modulating the transcription factor Sp1. We further established the roles of both CBS and SREBPs in regulating ovarian tumor growth *in vivo*. In orthotopic tumor models, CBS or SREBP silencing resulted in reduced tumor cells proliferation, blood vessels formation and lipid content. Hence, cancer-selective disruption of the lipid metabolism pathway is possible by targeting CBS and, at least for OC, promises a profound benefit.

## INTRODUCTION

Epithelial ovarian carcinoma (EOC) is the fifth most common cause of cancer deaths among women in Western Europe and the U.S [1]. Despite frequent initial responses to platinum/taxane therapy, most patients with advanced epithelial ovarian cancer (EOC) eventually develop drug resistance that leads to low responsiveness to any agent and shortened survival [2, 3]. A robust detection method based on molecular profiles for ovarian cancer has not yet been established because the disease exhibits metabolic changes due to the presence of the tumor and potential genetic variations that affect blood chemistry during the course of tumor progression [4, 5]. Hence, there is a critical need to device new therapeutic strategies to improve dismal prognosis in EOC.

Cancer cells require a constant supply of energy and structural components to support their proliferation and oncogenes actively reprogram metabolism to facilitate this supply [6]. In the last decade, the altered lipid metabolism has increasingly been recognized as another common property of malignant cells helping them achieve the overwhelming energy demand, providing membrane constituents and modulating signaling pathways [7]. A number of lipogenic enzymes utilize reduced nicotinamide adenine dinucleotide phosphate (NADPH) and acetyl-CoA generated from glucose and glutamine metabolism, to synthesize fatty acids and their derivatives [8]. Therefore, the exacerbated lipogenesis in cancer cells is not only caused by the upregulation of lipid metabolizing enzymes, but is also directly coupled to other common metabolic pathways and their associated cell signaling pathways [8]. Indeed deregulated lipid metabolism has been related to increases in the incidence of cancerous diseases, particularly breast, colorectal, ovarian, and prostate cancers [9, 10]. Lipids are important second messengers for cellular signaling and one such lipid of this class is phosphatidylinositol (3,4,5)-trisphosphate [PtdIns(3,4,5)*P*_3_; PIP_3_] [11]. Phosphatidylinositol (3,4,5)-trisphosphate is produced by PI3K in response to growth factor signaling and mediates the recruitment and activation of Akt [11]. Lipids are also involved in the post-translational modification of proteins such as palmitolyation, myristylotion, prenylation and geranylation, which affects the activity of several proteins including Hedgehog pathway [12]. Additionally aberrant lipid signaling promotes cell migration and involves diacylglycerol (DAG), lysophosphatidic acid (LPA) and prostaglandins [13]. Recently Nomura *et al*. reported a specific lipid signature that was associated with overexpression of monoglyceride-lipase (MAGL), a lipase involved in releasing free fatty acids (FFAs) from triacylglycerols (TAGs) and its overexpression was found in highly aggressive cancers and its inhibition caused defects in cancer cell migration and tumor growth [14]. Ovarian cancers have a clear predilection for metastasis to the omentum, an organ primarily composed of adipocytes [15]. A protein array identified upregulation of fatty acid–binding protein 4 (FABP4) in omental metastases as compared to primary ovarian tumors, and FABP4 expression was detected in ovarian cancer cells at the adipocyte-tumor cell interface, thus identifying an interesting connection between lipid metabolism and metastasis [16]. However, targeting lipid metabolism is challenging because multiple different feedback mechanisms, a multitude of metabolic enzymes having multiple isoforms with different metabolic functions, different cellular localization or tissue distribution, are involved and importantly they may be required for normal cellular functions [7]. Thus, cancer cell specific simultaneous targeting of multiple metabolic enzymes involved in lipid biogenesis is critical to improve therapeutic outcome and minimize side effects.

A landmark paper by Roth et al. demonstrated that pre-treatment with H_2_S prevented hypoxic injury in mice by drastic reduction of the animal's core body temperature [17], mimicking hibernation, a phenomenon dependent on lipid metabolism. While reports from Shatalin *et al*. deduced that H_2_S synthesizing enzymes such as cystathionine beta synthase (CBS) is a universal defense against antibiotics in bacteria and render them drug resistant [18]. We speculate that cancer cells might exploit similar mechanisms for metabolic rewiring and chemoresistance by involving CBS. Strikingly, CBS localizes in mice ovaries and is responsible for follicular development [19] suggesting the rationale of CBS involvement in ovarian cancer progression, as developmental pathways are reactivated in tumorigenesis [20]. Indeed our previous work showed for the first time the roles of CBS and thereby H_2_S in chemoresistance in ovarian cancer [21]. This function of CBS also involved mitochondrial localization of CBS and affected the ATP production and redox states, though role of CBS in lipid metabolism in cancer is still unexplored. Mice genetically deficient in CBS display abnormal lipid metabolism with markedly elevated triglyceride and nonesterified fatty acid levels in liver and serum [22]. The growth and body mass characteristics of CBS deficient *TgI278T Cbs*^−/−^ mice show that these animals have significantly decreased fat mass and total cysteine (tCys) compared to heterozygous sibling mice [22]. The decrease in fat mass was accompanied by a 34% decrease in liver glutathione (GSH) along with a significant decrease in liver mRNA and protein for the critical fat biosynthesizing enzyme Stearoyl CoA desaturase-1 (*Scd-1*) [22]. Further, elevated lipogenesis in epithelial stem-like cells have been shown to demonstrate apoptosis resistance in ductal carcinoma *in situ* of breast cancer [23]. Roles of several lipid metabolizing enzymes like phospholipaseA2, autotaxin, FAS and phospholipaseD have been implicated in ovarian cancer progression [24]. The aforesaid information is provocative and is suggestive of a probable role of CBS in regulating aberrant lipid metabolism in ovarian cancer.

The key regulation of lipid metabolism is attributed to the sterol regulatory element binding proteins (SREBPs) that are lipogenic transcription factors and critical links between oncogenic signaling and tumor metabolism [25]. The mammalian genome comprises of two distinct SREBP genes: *SREBP1* and *SREBP2* [26]. SREBP1 orchestrates lipogenic processes by activating genes involved in fatty acid and triglyceride biosynthesis, whereas SREBP2 transcribes genes involved in cholesterol synthesis [27]. Deregulated SREBP function has shown to be involved in pathological conditions like hepatic steatosis, type-2 diabetes, dyslipidemia and cancer [28].

Taken together the aforesaid cues, possible involvement of H_2_S synthesizing enzyme CBS in SREBP mediated or independent cancer metabolic reprogramming are undeniable. The present study reveals a novel molecular mechanism by which CBS promotes ovarian cancer growth and maintenance by involving SREBPs. We genetically manipulated CBS both *in vitro* and *in vivo* by siRNA and shRNA approach. Absence of CBS attenuated lipid content and SREBP expression as well as expression of its target genes. We show a role of CBS and SREBPs in cell proliferation, migration and invasion of ovarian cancer cells. Mechanistically CBS tunes SREBP expression by Sp1 mediated transcription. Silencing CBS and SREBPs significantly inhibit tumor growth in pre-clinical orthotopic mouse models. Taken together these results propose that CBS regulates lipid metabolism in ovarian cancer and provides a novel axis for ovarian cancer progression.

## RESULTS

### Ovarian cancer cell lines exhibit elevated lipid contents

**Figure 1:**
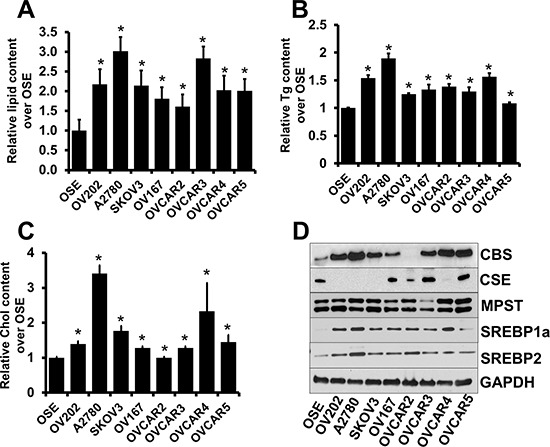
Aberrant lipid content in ovarian cancer cells **A.** Comparison of fold differences in lipid droplet content by Oil O Red staining between OSE and a panel of ovarian cancer cell lines per 30,000 cells. Values are means ± SD. *N* = 3. **B.** Comparison of fold differences of total triglyceride (Tg) content between OSE and a panel of ovarian cancer cell lines, as determined using Triglyceride Colorimetric Assay Kit (Cayman). Values are means ± SD. *N* = 3. **C.** Comparison of fold differences of total cholesterol (Chol) content between OSE and a panel of ovarian cancer cell lines, as determined using Cholesterol Fluorometric Assay Kit (Cayman). Values are means ± SD. *N* = 3. **D.** Expression of CBS, CSE, MPST, SREBP1a and SREBP2 in various ovarian cell lines as determined by immunoblotting. GAPDH is used as the loading control. For A–C, *P < 0.05 versus corresponding control.

Cancer cells frequently exhibit specific lipid metabolism reprogramming to support the rapid proliferation of cancer cells [29]. Recently, it was reported that cancer cells contain increased numbers of lipid droplets compared with normal tissue which are storage sites for triglycerides and cholesterol to be used as energy source [30]. We quantitated the lipid droplet content in various epithelial ovarian cancer (EOC) cell lines by Oil O Red staining and compared with that of ovarian surface epithelial (OSE) cells. Lipid droplet content was significantly higher across several ovarian cell lines suggesting a potential role of lipids in ovarian cancer maintenance and progression (Fig. 1A). Further we deduced the triglyceride and cholesterol content that acts as energy fuel, structural and signaling components [31], in these cell lines. We found that most of the ovarian cancer cell lines exhibited significantly high triglyceride and cholesterol content (Fig. 1B, 1C). These results indicate a role of aberrant lipogenesis in EOC growth. Our previous study identified roles of CBS in ovarian cancer maintenance and drug resistance [21], while the aberrant expression of SREBPs have been implicated in many forms of cancer [28, 32]. To further understand the role of CBS and SREBPs in human ovarian cancer, their expression level in normal ovarian vs. ovarian cancer cell lines were compared. We employed immunoblotting to assess the expression of CBS and SREBPs at the protein level (Fig. 1D). Minimal to no expression of CBS and SREBPs was observed in the non-malignant OSE cells. However, 7 out of 8 cancer cell lines expressed significantly high CBS, though the other H_2_S producing enzyme 3-mercaptopyruvate sulfurtransferase (MPST) and cystathionine gamma-lyase (CSE) did not reveal any significant difference with OSE (Fig. 1D), while 6 out of 8 cancer cell lines expressed significantly high SREBP1 or SREBP2 (Fig. 1D). Having confirmed that CBS and SREBPs are differentially overexpressed in ovarian cancer cell lines and possess significant lipid storage, we next sought to examine the functional significance and the correlation of CBS and SREBPs in ovarian cancer. Since P53 mutation is near universal in high grade serous ovarian cancer [33], we have chosen three different types of cancer cell lines having different p53 background to demonstrate functional significance of CBS and SREBPs in lipid metabolism in ovarian cancer, namely, A2780 (expresses only wild-type p53 [34]), SKOV3 (does not express p53 mRNA or protein [35]) and OVCAR4 (expresses mutated p53 [36]). Expressions of CBS and SREBPs are markedly higher in these cells as compared to normal OSE cell lines.

### CBS affects lipid content and lipid uptake in ovarian cancer cells

**Figure 2:**
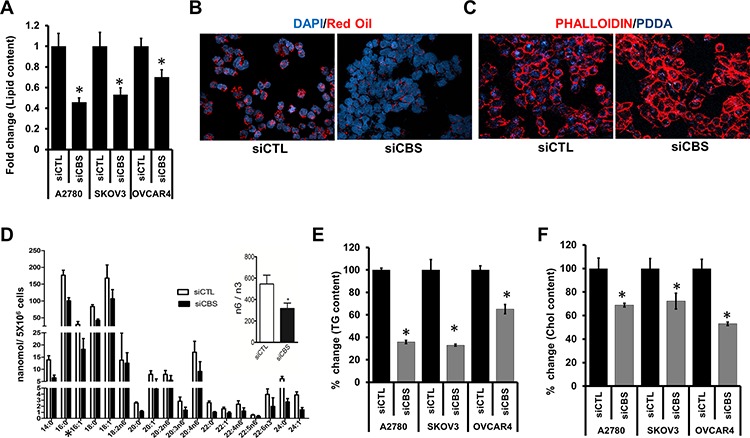
CBS affects lipid content and uptake in ovarian cancer cells **A.** Effect of CBS silencing (72 h post-transfection) on lipid droplet level in three different ovarian cancer cell line (A2780, SKOV3 and OVCAR4) determined by Oil O Red staining and absorbance quantified by spectrophotometer. Values are means ± SD. *N* = 3. **B.** Representative micrograph captured by confocal microscopy of siCTL- and siCBS- A2780 cells showing lipid droplets stained with Oil O Red. **C.** Effect of CBS silencing (72 h post-transfection) on lipid uptake. Representative micrograph of siCTL- and siCBS- A2780 cells showing fluorescent lipid (PDDA) uptake as captured by confocal microscopy. The cells are counter stained with Alexa Fluor 568-Phalloidin (red). **D.** Change in fatty acid levels in the cell lysates of scrambled siRNA (siCTL) and CBS siRNA (siCBS) treated A2780 cells measured by TLC/GC-FID. Ratiometric analysis of change in total fatty acid (n6/n3) in siCTL and siCBS cells (Inset). Values are means ± SD. *N* = 3. **E.** Effect of CBS silencing (72 h post-transfection) on the total triglyceride content of three cell lines (A2780, SKOV3 and OVCAR4). Comparison of fold differences of total triglyceride (Tg) content between siCTL and siCBS cancer cell lines. Values are means ± SD. *N* = 3. **F.** Effect of CBS silencing (72 h post-transfection) on the total cholesterol content of three cell lines (A2780, SKOV3 and OVCAR4). Comparison of fold differences of total cholesterol (Chol) content between siCTL and siCBS cancer cell lines. Values are means ± SD. *N* = 3. For A and D–F, *P < 0.05 versus corresponding control.

Accumulation of cytoplasmic lipid droplets (LDs) forms a basis of increased growth and chemoresistance in neoplastic cells [30]. To explore whether silencing CBS affects LD formation, we quantified the LDs by determining the absorbance of Oil O Red retained in three CBS silenced cell lines A2780, SKOV3 and OVCAR4, and observed a reduction by 55%, 47% and 30%, respectively (Fig. 2A). Furthermore, visualization of LDs by Oil O Red staining clearly showed a considerable decrease in LD number in siCBS- A2780 (Fig. 2B) as compared to control siCTL-A2780 group. Furthermore, cancer cells tend to uptake lipid from their microenvironment to meet their abnormally high energy demand [37]. To investigate whether intracellular uptake of lipids is also affected upon CBS silencing, we incubated siCTL-A2780 and siCBS-A2780 cells with fluorescently labelled fatty acid PDDA (1-pyrenedodecanoic acid) and monitored its uptake by confocal imaging. Absence of CBS abrogated the uptake of PDDA in siCBS-A2780 cells (Fig. 2C), indicating a role of CBS in lipid uptake in ovarian cancer cells. The lipogenic phenotype characterized by the activation of lipid metabolism is recognized as a universal feature of most cancers [7]. To determine the absolute fatty acid distribution in A2780 and their CBS silenced counterparts, we performed TLC/GC-FID to determine the content of each fatty acid. CBS silencing globally reduced the fatty acid components in the cell, affecting both saturated and unsaturated forms (Fig. 2D). The total fatty acid content was almost reduced by 50% upon silencing CBS (Inset Fig. 2D). Fatty acids synthesized in the cells are components for triglycerides and cholesterol [27]. CBS affected the total triglyceride content in the A2780, SKOV3 and OVCAR4 cell lines as the content of TG was reduced by 65%, 63% and 35%, respectively (Fig. 2E). Similarly the quantification of total cholesterol utilizing a cholesterol fluorometric assay kit (Cayman) in CBS silenced cells revealed a significant decrease of 32%, 28% and 47% in the A2780, SKOV3 and OVCAR4 cell lines, respectively (Fig. 2F). All of the above results indicate an important role of CBS in regulation of lipid metabolism in ovarian cancer.

### CBS regulates migration and invasion of ovarian cancer cells

**Figure 3:**
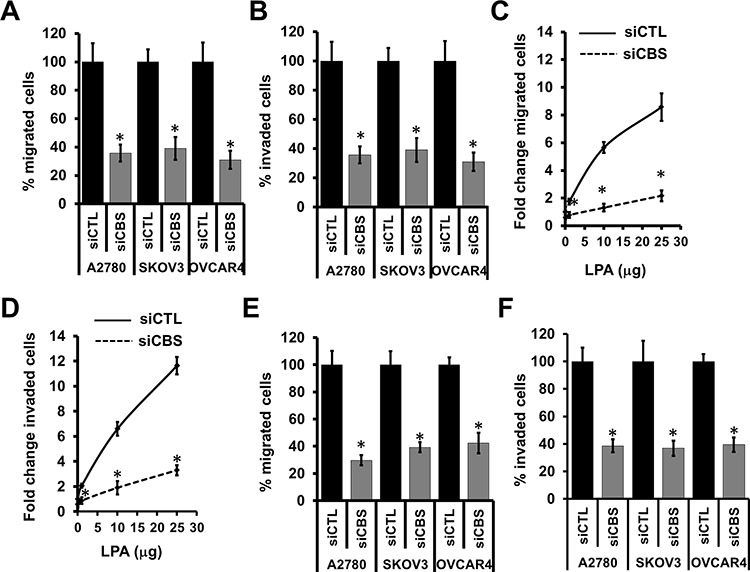
CBS regulates migration and invasion of ovarian cancer cells **A.** Silencing of CBS inhibits cell migration in ovarian cancer cells (A2780, SKOV3 and OVCAR4). Migration of siCTL and siCBS cells was examined using Boyden chamber. Cells were fixed and stained with crystal violet and counted under microscope. Percentage change values are means ± SD. *N* = 3. **B.** Silencing of CBS inhibits cell invasion of fibronectin matrix by ovarian cancer cells (A2780, SKOV3 and OVCAR4). Invasion of siCTL and siCBS cells through fibronectin-coated filters was examined using Boyden chamber. Cells were fixed and stained with crystal violet and counted under microscope. Percentage change values are means ± SD. *N* = 3. **C.** Silencing of CBS inhibits cell migration towards lipid (LPA) rich gradient. Migration of siCTL- and siCBS- A2780 cells towards increasing concentrations of LPA was examined using Boyden chamber. Cells were fixed and stained with crystal violet and counted under microscope. Percentage change values are means ± SD. *N* = 3. **D.** Silencing of CBS inhibits cell invasion of fibronectin matrix towards lipid (LPA) rich gradient. Invasion of siCTL- and siCBS- A2780 cells through fibronectin-coated filters towards increasing concentrations of LPA was examined using Boyden chamber. Cells were fixed and stained with crystal violet and counted under microscope. Percentage change values are means ± SD. *N* = 3. **E.** CBS silencing prevents the migration of ovarian cancer cells (A2780, SKOV3 and OVCAR4) towards LPA. Migration of siCTL and siCBS cells was examined using Boyden chamber. Cells were fixed and stained with crystal violet and counted under microscope. Percentage change values are means ± SD. *N* = 3. **F.** CBS silencing prevents the invasion of ovarian cancer cells (A2780, SKOV3 and OVCAR4) towards LPA. Invasion of siCTL and siCBS cells through fibronectin-coated filters towards increasing concentrations of LPA was examined using Boyden chamber. Cells were fixed and stained with crystal violet and counted under microscope. Percentage change values are means ± SD. *N* = 3. For A-F, *P < 0.05 versus corresponding control.

To provide further evidence for a role of CBS in ovarian cancer pathophysiology, we next investigated a role of CBS in ovarian cancer cell migration and invasion. We transiently silenced ovarian cancer cell lines with siRNA of CBS and compared with scrambled controls. A2780 cells transfected with scrambled control (siCTL-A2780) migrated efficiently toward an FBS gradient (Fig. 3A), whereas silencing of endogenous CBS expression resulted in a marked decrease in the cell migration (Fig. 3A). Similar results were obtained in SKOV3 and OVCAR4 cells (Fig. 3A). Quantitation of results indicated that the silencing of CBS attenuated the A2780 cell migration by 62%, SKOV3 cells by 59%% and OVCAR4 cells by 65% (Fig. 3A). CBS silencing, however, showed no significant decrease in cell proliferation during the course of the migration study, confirming that the decrease in cell migration upon CBS silencing is due the effect on cell migratory pathways and not due a decrease in cellular proliferation (Fig. S1A). We also examined whether CBS affects the cellular invasion in ovarian cancer. A significant decrease in invasion of A2780, SKOV3 and OVCAR4 cells was observed when these cells were transiently silenced by CBS siRNA as compared to scrambled control. (Fig. 3B). Quantitation of invading cell numbers indicated that downregulation of CBS expression reduced invasion of siCBS-A2780 by 65%, siCBS-SKOV3 cells by 61% and siCBS-OVCAR4 cells by 70% (Fig. 3B). Ovarian cancer cells tend to migrate to lipid rich gradient such as the omentum [15]. We utilized the LPA gradient as chemoattractant to monitor the cell migration of A2780 cells in the presence and absence of CBS. While control cells efficiently migrated towards the LPA rich environment showing a dose response, CBS silencing blunted such events (Fig. 3C). Similarly LPA induced a dose response in cell invasion properties of A2780 cells, while silencing of CBS abrogated the phenotype (Fig. 3D). Absence of CBS prevented the cancer cells to migrate or invade in a LPA gradient (Fig. 3E, 3F), indicating a role of CBS promoting migration and invasion of ovarian cancer cells towards lipid rich environment.

### CBS regulates the expression of SREBPs and its target genes

**Figure 4:**
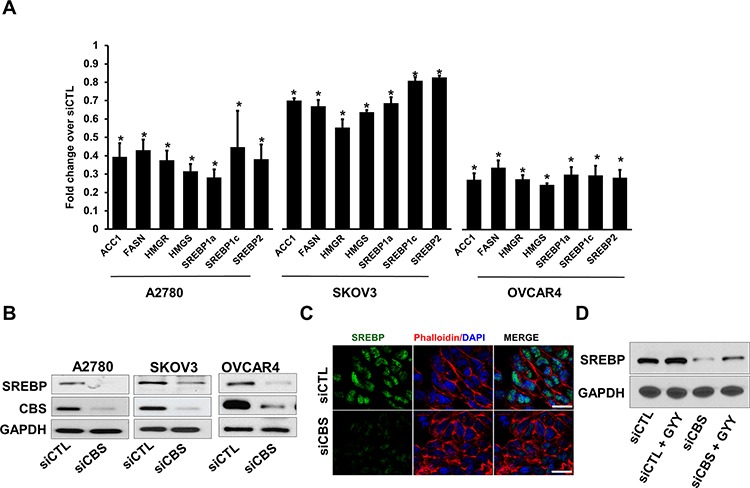
CBS silencing reduces expression of lipogenic genes and SREBPs in ovarian cancer cells **A.** Expression profile (qRT-PCR) of fatty acid and triglyceride biosynthesis genes in ovarian cancer cell lines (A2780, SKOV3 and OVCAR4) post CBS silencing, relative to cells transfected with, relative to cells with siCTL. **B.** Western blot analysis of the CBS and SREBP proteins in siCTL cells or cells with silencing of CBS (siCBS). GAPDH is used as the loading control. **C.** CBS silencing prevents nuclear translocation of SREBP. The fixed siCTL- and siCBS- A2780 cells were stained using Anti-SREBP antibody (1:100) followed by Alexa Fluor 488-conjugated secondary antibody. Then the localization of SREBP was visualized by confocal microscopy. **D.** Immunoblot for SREBP showing recovered expression in CBS silenced cells upon GYY4137 treatment (1 mM, 24 h). GAPDH is used as the loading control. For A, *P < 0.05 versus corresponding control.

It is well-known that lipid metabolism is tightly regulated, particularly at the level of transcription (e.g. by SREBP and LXR) [38]. SREBPs are transcription factors that transcribe target genes involved in lipid biogenesis [28]. The SREBPs are controlled at multiple levels, such as the regulation of mRNA expression, cellular localization or post-transcriptional modification by nutrient-dependent signaling pathways (e.g., insulin) [25]. Intriguingly CBS deficient mice models exhibit abnormal fat loss [22]. To investigate a functional link between CBS and three OC cell lines A2780, SKOV3 and OVCAR4, we transiently transfected the cells with CBS siRNA and probed for transcriptional regulation of *SREBP*. All cell lines demonstrated an appreciable downregulation of the gene expression of individual SREBP isoforms *SREBP1a, SREBP1c* and *SREBP2* along with their established target genes like *ACC1, FASN, HMGR* and *HMGS*. (Fig. 4A). Furthermore, to rule out the possibility of off target effects, we also used another siRNA targeting a different region of CBS mRNA and obtained similar effects on the gene expressions of SREBPs and their target genes (Fig. S1B), thus excluding the possibility of non-specific siRNA effects. CBS silencing resulted in significant reduction in the expression of SREBP at the protein levels as well (Fig. 4B), suggesting a regulatory effect of CBS in lipid metabolism pathway in ovarian cancer. Furthermore, treatment of A2780 cells with a small molecule inhibitor of CBS enzymatic function, aminooxyacetic acid (AOAA), resulted in diminished expression of SREBPs and their target genes (Fig. S1C, S1D). Inactive SREBPs are cytosolic proteins associated with the ER and is processed to transcriptionally active form that translocated to the nucleus, by two proteases S1P and S2P at the Golgi apparatus [39]. We probed for the presence of active SREBP in the nucleus of A2780 cells transfected with scrambled siRNA or CBS siRNA. Silencing of CBS resulted in profound loss of SREBP in the nucleus corroborating the observation of attenuated expression of SREBPs (Fig. 4C). CBS is a metabolic enzyme that yields H_2_S gas as a byproduct [40]. To determine if H_2_S could rescue SREBP expression, A2780 cells were exposed to synthetic H_2_S donor GYY4137 which led to an increase in SREBP expression (Fig. 4D), even in CBS silenced background. Taken together the results indicate that CBS regulates lipid metabolism in ovarian cancer cells via regulation of SREBP expression, translocation and transcriptional activity.

### SREBPs regulate proliferation, migration and invasion of ovarian cancer cells

**Figure 5:**
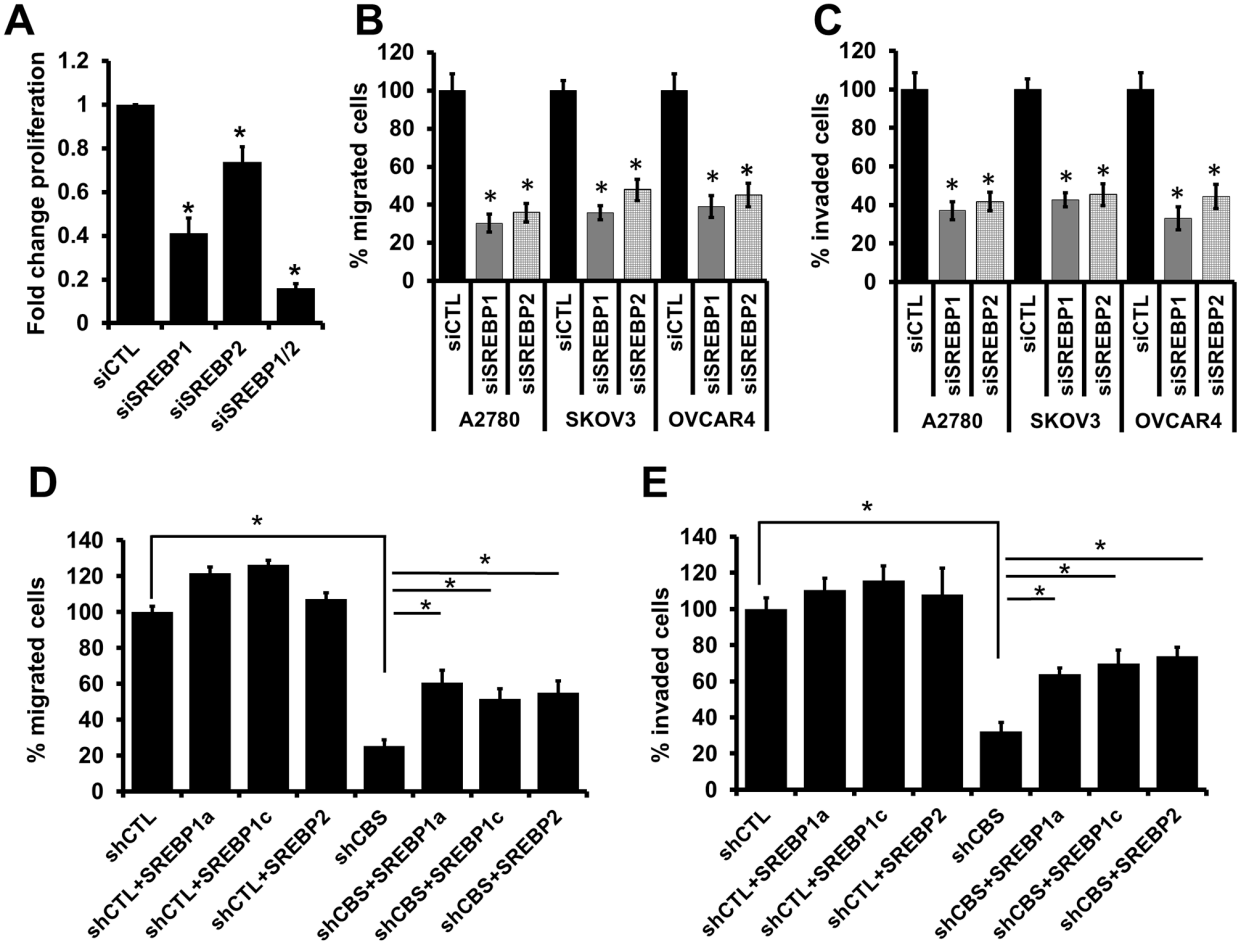
SREBPs regulate ovarian cancer cell proliferation, migration and invasion **A.** Effect of gene silencing of SREBP1, SREBP2 or both on A2780 cell proliferation. Fold change proliferation values are means ± SD. *N* = 3. **B.** Silencing of SREBP1 and SREBP2 inhibits cell migration in ovarian cancer cells (A2780, SKOV3 and OVCAR4). Migration of siCTL and siCBS cells was examined using Boyden chamber. Cells were fixed and stained with crystal violet and counted under microscope. Percentage change values are means ± SD. *N* = 3. **C.** Silencing of SREBP1 and SREBP2 inhibits cell invasion of fibronectin matrix by ovarian cancer cells (A2780, SKOV3 and OVCAR4). Invasion of siCTL and siCBS cells through fibronectin-coated filters was examined using Boyden chamber. Cells were fixed and stained with crystal violet and counted under microscope. Percentage change values are means ± SD. *N* = 3. **D.** Overexpression of SREBP1 or SREBP2 can restore migration phenotype in CBS silenced cells. Transient transfection of Flag tagged -SREBP1a, -SREBP1c and -SREBP2 induces cell migration in CBS knockdown A2780 cells (shCBS). Migration of cells was examined using Boyden chamber. Cells were fixed and stained with crystal violet and counted under microscope. Percentage change values are means ± SD. *N* = 3. **E.** Overexpression of SREBP1 or SREBP2 can restore invasive properties in CBS silenced cells. Transient transfection of Flag tagged -SREBP1a, -SREBP1c and -SREBP2 induces cell invasion through fibronectin-coated filters in CBS knockdown A2780 cells (shCBS). Invasion of cells was examined using Boyden chamber. Cells were fixed and stained with crystal violet and counted under microscope. Percentage change values are means ± SD. *N* = 3. For A-F, *P < 0.05 versus corresponding control.

To further investigate whether CBS-regulated SREBPs expression is responsible for proliferation, migration and invasion of ovarian cancer cells, we transiently silenced ovarian cancer cell lines with siRNA for SREBP1 and SREBP2 (Fig. S1E). The proliferation of A2780, SKOV3 and OVCAR4 cells were significantly reduced upon SREBP1 and SREBP2 silencing (Fig. 5A), while concomitant silencing of SREBP1 and SREBP2 resulted in drastic attenuation of cell proliferation (Fig. 5A). To further understand the effects of silencing of SREBPs on migration of ovarian cancer cells, Boyden chamber migration assay was performed. Control A2780, SKOV3 and OVCAR4 cells migrated efficiently toward an FBS gradient (Fig. 5B), and the silencing of endogenous SREBP1 expression resulted in a marked decrease in the cell migration (Fig. 5B). Similar results were obtained in all the cell lines with SREBP2 silencing (Fig. 5B). Quantitation of results indicated that the silencing of SREBP1 attenuated the A2780 SKOV3 and OVCAR4 cell migration by 70%, 65% and 61% respectively (Fig. 5B). Similarly SREBP2 silencing resulted in 65%, 53% and 55% in A2780, SKOV3 and OVCAR4 cells, respectively (Fig. 5B). These results support the idea that SREBPs are required for the directional migration of ovarian cancer cell. Quantitation of invading cell numbers indicated that decreased expression of SREBP1 reduced invasion of siSREBP1-A2780 by 63%, siSREBP1-SKOV3 cells by 58% and siSREBP1-OVCAR4 by 67% (Fig. 5C) as compared to the scrambled control cells. Similarly, silencing of SREBP2 also resulted in a significant decrease in the cell invasion of the above three cell lines (Fig. 5C). Importantly, SREBP silencing did not alter cellular proliferation throughout the duration of migration and invasion experiments (Fig. S1F). Therefore, the above results indicate that SREBPs play critical role in the migration and invasion of ovarian cancer cells.

### SREBPs can restore ovarian cancer cell migration and invasion in the absence of CBS

We determined above that CBS silencing downregulate expression of SREBPs and also abrogate cellular motility. We further attempted to realize whether restoring SREBPs in a CBS knockdown milieu could rescue the migratory and invasive phenotype of ovarian cancer cells. For this purpose, we depleted endogenous CBS by stable knockdown using lentiviral approach and transfected cells with Flag-SREBP1a, Flag-SREBP1c and Flag-SREBP2. We confirmed the efficiency of knockdown of CBS (Fig. S1G) and overexpression of SREBPs by determining the respective proteins by immunoblotting (Fig. S2A). Knockdown of CBS in A2780 and OVCAR4 cells did not affect the expression of cystathionine gamma-lyase (CSE), another enzyme that also feeds into the sulphur metabolizing enzymatic pathway (Fig. S1G).

SREBP overexpression did not alter the CBS expression levels neither in shCTL- nor shCBS- A2780 cells (Fig. S2B). In shCTL-A2780 cells, forced overexpression of SREBPs did not impact the cell migration (Fig. 5D), suggesting these cells express sufficiently high levels of endogenous SREBPs to control the cell migration. However, forced overexpression of SREBP1a, SREBP1c and SREBP2 in CBS silenced cells could partially restore migratory and invasive behavior of all the three ovarian cancer cell lines tested (Fig. 5D, 5E), establishing a direct link between CBS and SREBPs in cellular motility in ovarian cancer. Moreover the invasive property of these cells were similarly modulated by CBS silencing or SREBP overexpression in matrigel matrix (Fig. S2C, S2D).

### Regulation of SREBPs by CBS

**Figure 6:**
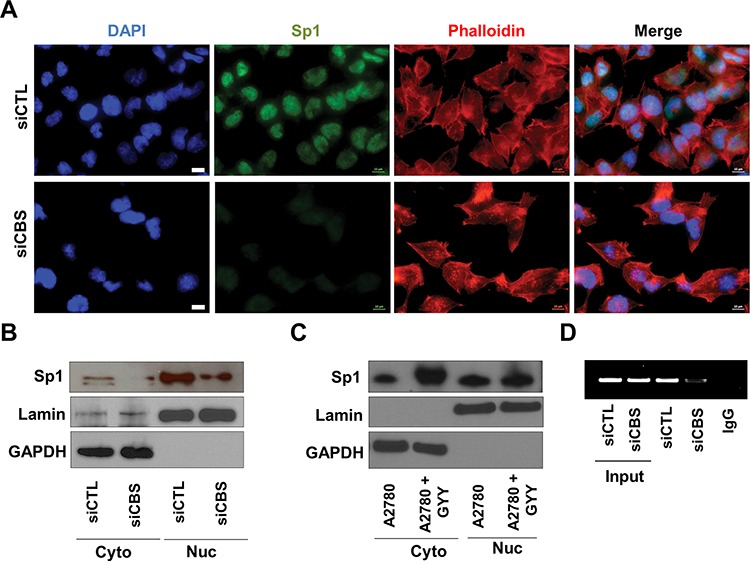
Regulation of SREBPs by CBS **A.** CBS silencing prevents nuclear translocation of Sp1. The fixed siCTL- and siCBS- A2780 cells were stained using Anti-Sp1 antibody (1:500) followed by Alexa Fluor 488-conjugated secondary antibody and Alexa Fluor 568-Phalloidin incubation. Then the localization of Sp1 was visualized by immunofluorescence. **B.** Immunoblotting data exemplifying the effect of CBS silencing on the translocation of Sp1 in A2780 cells. Lamin and GAPDH were used for loading controls for nuclear and cytosolic fractions respectively. **C.** Immunoblotting data exemplifying the effect of GYY4137 on the translocation of Sp1 in A2780 cells. Lamin and GAPDH were used for loading controls for nuclear and cytosolic fractions respectively. **D.** ChIP assay reveals CBS silencing abrogates Sp1 binding on the promoter of SREBP.

To investigate the transcriptional regulation of SREBPs by CBS, we analyzed the probable transcription factors for SREBPs and identified Sp1 as a putative candidate [41]. Indeed Sp1 nuclear translocation was hindered upon CBS silencing as visualized by immunofluorescence (Fig. 6A). Similar effects of CBS silencing on Sp1 translocation was observed by cell fractionation followed by western blotting (Fig. 6B), while a H_2_S donor, GYY4137, increased the translocation of Sp1 to the nucleus (Fig. 6C). To conclusively determine the role of CBS in Sp1 driven SREBP transcription, we analyzed binding of Sp1 on SREBP promoter by ChIP. CBS silencing results in significantly low promoter binding by Sp1 (Fig. 6D), which explains transcriptional control of SREBPs by CBS.

### CBS and SREBPs promote tumor growth *in vivo*

**Figure 7:**
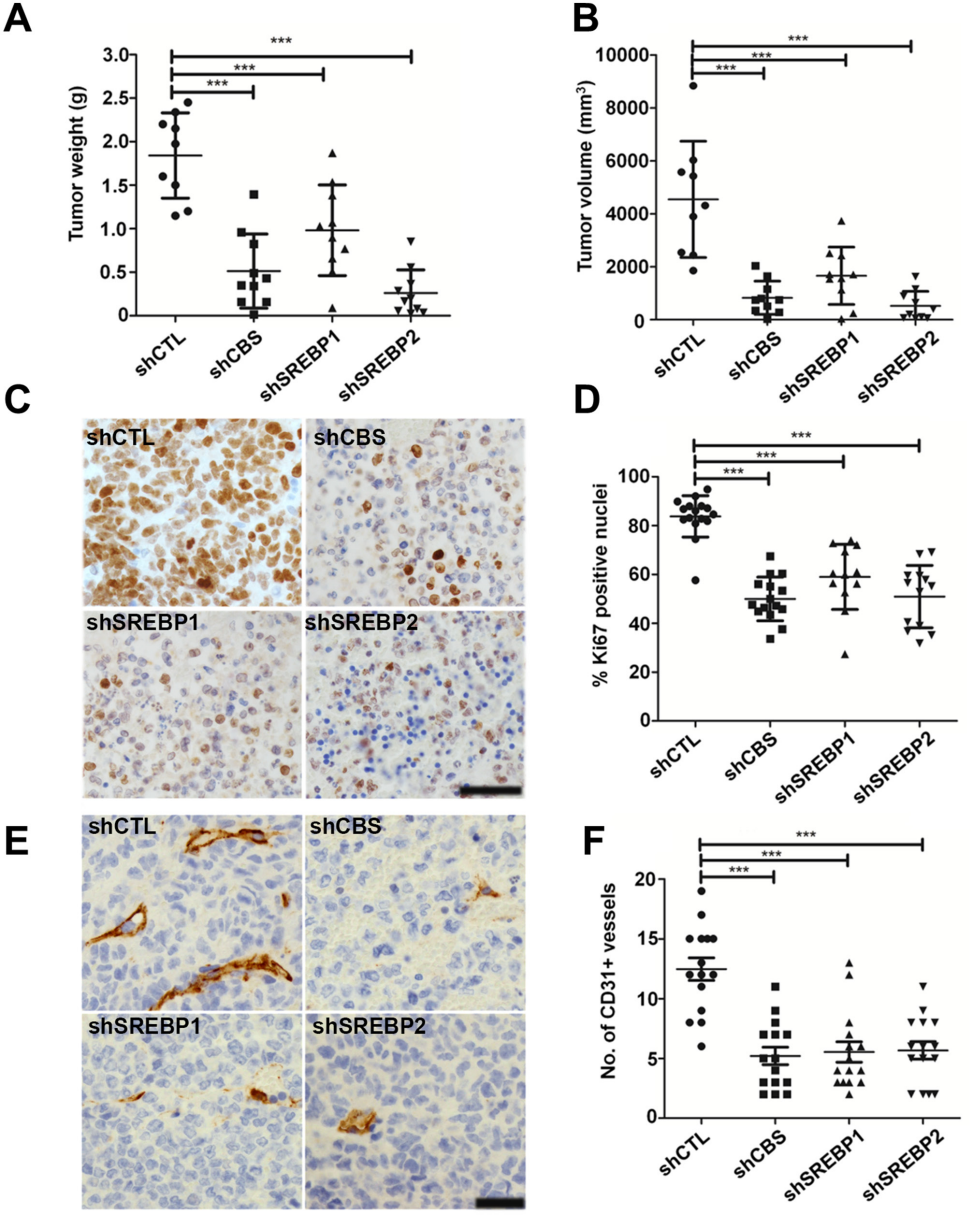
Effect of CBS and SREBPs knockdown on orthotopic ovarian cancer growth **A.** Knockdown of CBS, SREBP1 and SREBP2 inhibits tumor growth. The shCTL-, shCBS-, shSREBP1 and shSREBP2-A2780 cells (1.5 × 10^6^) were orthotopically implanted in both the ovaries of athymic nude mice. Animals were sacrificed 3 weeks after implantation and ovaries were removed, weighed, measured and photographed. Scatter plot shows the tumor weights from individual animals in each group. **B.** Knockdown of CBS, SREBP1 and SREBP2 decreases tumor volume. **C.** Representative histology of tumors from mice xenografts of A2780-shCTL, -shCBS, -shSREBP1 and shSREBP2 cells with Ki67. **D.** Quantification of Ki67 staining shows a notable reduction in tumors from shCBS, -shSREBP1 and -shSREBP2 compared to the shCTL group. **E.** Representative histology of tumors from mice xenografts of A2780-shCTL, -shCBS, -shSREBP1 and -shSREBP2 cells with CD31. **F.** Quantification of CD31 staining analysis showed a remarkable reduction in vessel formation in tumors from shCBS, shSREBP1 and shSREBP2 compared to the shCTL group. Each group contains 9~10 mice. For A, B, D and F *P < 0.05 versus corresponding control.

**Figure 8:**
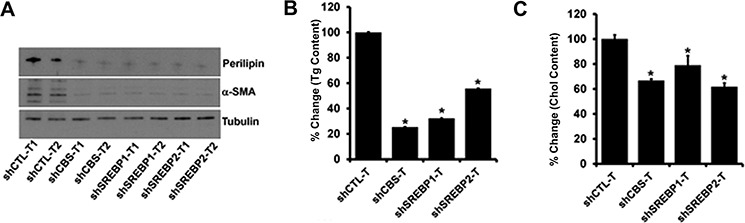
A. Immunoblot analysis of a-SMA and Perilipin in protein lysates isolated from tumors **B.** Effect of CBS, SREBP1 and SREBP2 knockdown on the total triglyceride content of tumors. Comparison of fold differences of total triglyceride (Tg) content between shCTL and shCBS, shSREBP1 and shSREBP2 tumors. Values are means ± SD. *N* = 2. **C.** Effect of CBS, SREBP1 and SREBP2 knockdown on the total Cholesterol content of tumors. Comparison of fold differences of total triglyceride (Tg) content between shCTL and shCBS, shSREBP1 and shSREBP2 tumors. Values are means ± SD. *N* = 2. For B and C *P < 0.05 versus corresponding control.

We examined the role of CBS and SREBPs in ovarian tumor growth. To provide evidence for the aforesaid role of CBS, we generated ovarian cancer cell lines with stable knockdown of CBS by lentiviral approach. Recently, the epithelium of the fallopian tube fimbriae (FTE) has been proposed to be origin of high-grade serous cancers (HGSC) [42, 43]. However, Matzuk group has also demonstrated recently that the ovarian surface epithelial cells could also be the site of origin for HGSC in mice, as even after the removal of fallopian tubes in TKO mice, ovaries alone developed metastatic HGSCs [44]. Since CBS functions independent of P53 status, we chose A2780 cell line that has functional TP53 as a model for TP53- mutation independent ovarian cancer model [34]. Rodents have a unique bursal membrane that surrounds the ovary and is continuous with the oviduct, which facilitates orthotopic injection of cancer cells leading to tumors in intrabursal space [45]. Furthermore, previously we have reported that A2780 cells form large tumors after orthotopic implantation into the ovarian bursa [46]. A2780 cells with or without CBS or SREBP-1 and -2 knockdowns were orthotopically injected in the ovaries of athymic nude mice. shCTL-A2780 cells formed tumors within 3 weeks of implantation and the average tumor weight was 1.8 g (Fig. 7A). Stable knockdown of CBS significantly inhibited the tumor growth and the average weight of these tumors was 0.6 g (Fig. 7A). Similarly stable knockdown of SREBP-1 and -2 significantly inhibited the tumor growth and the average weight of these tumors was 1.0 g and 0.2 g, respectively (Fig. 7A). Similarly, the tumor volumes corroborated with the tumor weight data. The tumor volumes were significantly reduced in tumors from shCBS, shSREBP1 and shSREBP2 cells (Fig. 7B). Silencing of CBS resulted in −50% decrease in Ki67 (a proliferation marker) positive cells compared with the shCTL group (Fig. 7C, 7D). While mice injected with shSREBP1 cells showed a 25% reduction in Ki67-positive cells, whereas shSREBP2 group showed a 30% reduction (Fig. 7C, 7D). Mice bearing tumors from shCBS, shSREBP1 and shSREBP2 cells also revealed a substantial decrease in number of blood vessels as determined by CD31 staining (Fig. 7E, 7F). To further investigate the effect of CBS and SREBP silencing in lipogenesis *in vivo*, we analyzed the expression of lipid droplet associated protein (Perillipin) [47] and α-SMA in the tumors from control and/or CBS and SREBPs knockdown cells. We found a significant loss of perilipin and α-SMA in the tumors from CBS and SREBP knockdown cells (Fig. 8A). In addition, we determined the total triglyceride and cholesterol content in the tumor specimens and identified a substantial decrease of the biomolecules in CBS and SREBPs knockdown tumors (Fig. 8B, 8C). Taken together our *in vivo* results indicate a potential role of CBS and SREBPs, in aberrant lipogenesis in ovarian cancer that promotes tumor growth.

## DISCUSSION

An enhanced rate of lipid synthesis in cancerous tissues has long been recognized as an important aspect of the rewired metabolism of transformed cells [7, 12]. Though expression of CBS has been recently reported to promote tumorigenesis in ovarian and colon cancer [21, 48], with minimal to no expression in OSE cells, the role of CBS was never been elucidated in altered lipid metabolism of cancer cells so far. Many genes coding for enzymes involved in FA and cholesterol biogenesis are targets of the sterol regulatory element-binding proteins (SREBPs), a family of transcription factors that are crucial for maintaining cellular lipid homeostasis [49]. Aberrant activation of SREBPs can contribute to obesity, fatty liver disease and insulin resistance, and could also be involved in cancer development [25, 50]. Indeed we found elevated expressions for all the isoforms of SREBP in most of the ovarian cancer cell lines (Fig. 1D). This correlated with similar pattern of expression for the H_2_S generating enzyme CBS (Fig. 1D). The other two enzymes involved in H_2_S generation in cells, CSE and MPST, did not show differences in expression between normal OSE cells and the battery of ovarian cancer cell lines. Ample evidence exists that fat animals are more likely to develop cancer than lean animals [51]. Tumors in fat animals also grow faster and larger, spread more quickly, and are more resistant to treatment [52]. In a landmark 2003 study, American Cancer Society researchers analyzed data on obesity and cancer and found that the most obese women had a 62% increase in their risk of dying from cancer than women of normal weight; for obese men, the increase was 52% [53]. Similarly an analysis including information on tumor characteristics of ovarian cancer and breast cancer done in the Swedish AMORIS database provided more insight into possible links between lipid metabolism and the risk of these cancers [54]. Taken together the aforesaid cues and the observation of fat loss in CBS knockout mice [22] bolsters the notion of CBS being involved in regulating lipid metabolism in ovarian cancer.

Increasingly, lipidomics approaches are being applied to analyze the lipid composition of cancer cell lines or fresh tumor specimens by mass spectrometry [55]. Positron emission tomography (PET) with acetate or choline tracers is used to visualize active lipid synthesis in tumors and could be used for the dynamic monitoring of response to treatments targeting lipid metabolism [56]. Our TLC/GC-FID approach revealed CBS regulates the expression of most FAs in ovarian cancer (Fig. 2D). In addition, because cancer cells might be able to obtain some rate-limiting lipids through uptake from the surrounding tissue, it is also essential to identify the lipid composition of the tumor microenvironment [37]. Indeed the aggressive “triple-negative” breast cancer cell lines express lipoprotein lipase (LPL), the key enzyme for extracellular lipolysis, and the transmembrane channel for exogenous free FA uptake (CD36), together with the classical lipogenic markers such as FASN [57]. Additionally, lipolysis-derived FAs may be able to attenuate any therapeutic advantage achieved by targeted inhibition of *de novo* FA synthesis [58]. In selected cancer cell lines lipolysis is, therefore, an additional pathway for FA acquisition and our data suggest CBS regulates lipid uptake from its surroundings (Fig. 2C). Recently, the omental adipocytes have shown to promote ovarian cancer cell homing to the omentum through the induction of specific, adipocyte-derived cytokines (adipokines) while ovarian cancer cells activate lipolysis within the adipocytes and use adipocyte-derived lipids for β-oxidation, thereby obtaining energy for tumor growth [16]. We observed that the expression of CBS in cancer cells induces an affinity for lipids and ovarian cancer cells effectively migrated and invaded towards a lipid gradient. These observations purport targeting CBS could plausibly abrogate the omental metastasis.

CBS generates a reducing environment through H_2_S that could activate the Hypoxia Inducible Factors (HIFs) [59]. It was demonstrated that HIF1 induces the expression of FASN in human breast-cancer cell lines and that FASN expression is increased in hypoxic tumor regions [60]. Because the flow of carbon from glucose to fatty acids is attenuated by hypoxia, other carbon sources are required to support fatty-acid synthesis under these conditions [31]. Indeed, acetyl-CoA synthetase 2, the bidirectional enzyme catalyzing the synthesis of acetyl-CoA from cytoplasmic acetate, is induced by hypoxia and promotes cancer-cell survival under these conditions [60]. More recently, three independent studies [61–63] showed that glutamine becomes the major carbon source for lipid synthesis in the absence of functional mitochondria. These studies found that isocitrate dehydrogenase-1 can produce cytoplasmic citrate by reductive carboxylation of glutamine-derived α-ketoglutarate [62, 63]. These posit the coupling of CBS, an amino acid metabolizing enzyme to the regulation of lipid metabolism. Indeed sulphur-containing amino acids (SAAs) like cysteine are recognized to be some of the most potent modulators of lipid metabolism [64] among amino acids but the molecular mechanisms are far from clear and could be additional effects from cysteine metabolism involving CBS.

Hydrogen sulfide (H_2_S) has recently emerged as a mammalian gaseous messenger molecule, akin to nitric oxide and carbon monoxide and is predominantly formed from Cys or its derivatives by the enzymes CBS and CSE [65]. One of the mechanisms by which H_2_S signals is by sulfhydration of reactive Cys residues in target proteins like GAPDH [66], Parkin, NFκB and others [67]. Since CBS regulates intracellular GSH and H_2_S levels and functions of SREBPs are known to be regulated by post translational modifications (PTMs) such as phosphorylation [68] and acetylation [69], novel PTMs such as glutathionylation and sulfhydration of SREBPs could be a factor impacting aberrant lipogenesis required for tumor growth, metastasis and drug resistance in EOC.

We identified CBS silencing regulates SREBPs through Sp1 at the expression levels respectively. Indeed A'lvarez *et al*. have mapped the minimal human SREBP-1a promoter region to 75 bp upstream of the translation start site and discovered a functional role for the 3 GC-boxes containing overlapping sites for the Sp1 and EGR-1 transcription factors [70]. Intact Sp1-binding sites are essential for promoter activity and strikingly we identified CBS silencing affects Sp1 binding to the promoter site of SREBP-1a plausibly through negating the nuclear translocation of Sp1 or affecting Sp1 protein stability, which is maintained by sulfhydration in presence of CBS (unpublished data). Taken together the role of CBS in regulation of SREBPs, introduces CBS as an important player to the field of lipid metabolism in cancer.

Effective targeting of lipid metabolizing enzymes has shown promise for cancer prevention and therapy [71]. FASN inhibitors have been shown to be effective in chemoprevention of breast cancer in *HER2/neu* transgenic mice, while other enzymes within the FA biosynthesis pathway have also been targeted experimentally and were shown to limit the growth and proliferation of cancer cells; such enzymes include ACC and SCD [72]. Moreover, silencing of ACLY has been shown to block cancer cell growth both *in vivo* and *in vitro* [73]. SB-204990, an inhibitor of ACLY that has been shown to lower hepatic cholesterol and FA synthesis rates in rats, also reduced tumor formation in lung and prostate xenografts [74]. Nevertheless, the chemical inhibition of the FA synthesizing enzymes shows severe side effects in animal models, including dramatic weight loss and psychological imbalance [72]. Additionally, lipolysis-derived FAs uptake may be able to attenuate any therapeutic advantage achieved by targeted inhibition of *de novo* FA synthesis [58]. Simultaneous targeting of multiple lipid metabolism routes in cancer holds potential for effective therapy in ovarian cancer. Recently, Tebbe *et al*. has shown metformin could inhibit adipogenesis by inhibition of key adipogenesis regulating transcription factors (CEBPα, CEBPΔ, and SREBP1), and induced AMPK, and thereby could be a therapeutic option for ovarian cancer at an early stage, as it not only targets ovarian cancer, but also modulates the environmental milieu [75]. In this scenario targeting CBS can exert dual decimation of both lipid synthesis and lipid uptake. While Platinum-based chemotherapy is the mainstay of care for multiple types of cancer, including ovarian cancer, the disease almost always returns and develops drug resistance [2]. Recently, Roodhart et al. demonstrated endogenous mesenchymal stem cells (MSCs) become activated during treatment with platinum analogs and secrete two distinct platinum-induced polyunsaturated fatty acids (PIFAs), KHT and 16:4(n-3), that in minute quantities induce resistance to a broad spectrum of chemotherapeutic agents [76]. CBS ablation causes normalization of most of the fatty acids and this property of CBS can be exploited to overcome platinum resistance. Hence we propose a therapeutic opportunity in ovarian cancer by selective targeting of CBS that orchestrates fueling of cancer cells by aberrant lipid metabolism.

## EXPERIMENTAL PROCEDURES

### Reagents, cell lines and culture

The following antibodies were used for immunoblotting: Rabbit polyclonal CBS antibody (H-300, sc-67154, Santa Cruz), rabbit polyclonal CTH/CSE antibody (Proteintech, 12217-1-AP), mouse monoclonal β-Actin antibody (Sigma, A-2228), rabbit polyclonal to Sp1 (Millipore, 07–645), rabbit polyclonal SREBP-1 antibody (C-20, sc-366, Santa Cruz), rabbit polyclonal SREBP-2 antibody (Cayman Chemical, 10007663), rabbit polyclonal to GAPDH (Sigma G9545) and anti-α-tubulin (Abcam, ab4074). PDDA (1-pyrenedodecanoic acid) was purchased from Invitrogen (P96). siRNA against human CBS were procured from two different sources (SI02777159 (QIAGEN) and (SASI_Hs01_00214623, Sigma)) and scrambled control siRNA (1027280) was from QIAGEN, CA, USA. CBS inhibitor aminooxyacetic acid (AOAA) was procured from MP Biomedicals, LLC (204159). Cell culture media RPMI-1640 and 0.25% Trypsin-EDTA were from Lonza. MCDB105 and Medium199 were from Sigma-Aldrich. Lipofectamine 2000 and Optimem-I were from Invitrogen. A2780 and OVCAR4 cell lines (Sigma-Aldrich) were routinely cultured in RPMI (Corning) supplemented with 10% heat inactivated FBS (Fisher Scientific) and 100 u penicillin-100 μg streptomycin/ml medium (Invitrogen) in a 5% CO_2_ humidified atmosphere. SKOV3 cell line from ATCC were grown in McCoy's 5A media supplemented with 10% FBS and 1% antibiotic. OSE (obtained from V. Sridhar, Mayo Clinic) was cultured in a 1:1 Media 199: MCDB 105 with 100 u penicillin-100 μg streptomycin/ml and 15% heat-inactivated serum.

### Plasmids, constructs and siRNA transfection

Gene silencing was performed in 6 cm culture dish containing 5 × 10^5^ cells in suspension using Hiperfect (Qiagen) and 10 μM siRNA (scrambled control siRNA (1027280), QIAGEN, CA, USA; CBS siRNA (SAS1-HS01–00214623, Sigma) and SREBP siRNA SAS1-HS01–00228542, Sigma) in OPTIMEM (Invitrogen). Effective silencing was achieved after 72 h of transfection (determined by protein expression) and all experiments with gene silencing were performed 48–96 h post transfection. Transfection of A2780 with SREBP1a- Flag, SREBP1c-Flag, SREBP2-Flag or Empty vector was performed using Lipofectamine 2000 with 1 μg plasmid DNA. Cells were incubated for 24 h after transfection prior to testing for transgene expression or performing downstream experiments.

### Proliferation assays

Transfected A2780 cells were collected by trypsinization after 48 of transfection, counted and seeded in 96-wellplates (2.5 × 10^3^ cells/well) and cultured for 24 h. Cell proliferation was determined using the CyQUANT^®^ NF Cell Proliferation Assay Kit (Invitrogen, C7026) according to the manufacterers protocol and fluorescence intensity was measured at excitation at 485 nm and emission detection at 530 nm. Experiments were repeated at least three times each time in triplicate.

### Oil red O staining

Cells (30,000) were seeded on a coverslip in a 24-well plate and were grown for 24 hours in the presence of complete growth medium. Cells were washed and fixed in 4% paraformaldehyde for 10 min at room temperature followed by further incubation with 4% paraformaldehyde for at 1 hour. Cells were washed with 60% isopropanol for 5 min at RT and then rinsed with water and air dried followed by staining with Oil Red O working solution and incubated at RT for 10 min. Coverslips were washed with ddH2O and mounted in a slide with Prolong Gold Antifade Reagent (Invitrogen). Oil Red O stained cells were examined under inverted confocal fluorescence microscope (Olympus, FV500). For quantification Oil Red O dye was eluted by adding 100% isopropanol and incubate for 10 min with gently shaking. The absorbance of the eluted dye was measured at 500 nm using 100% isopropanol as blank.

### Fatty acid analysis

Cellular lipids from frozen A2780 cells were extracted by a modified Bligh–Dyer technique in the presence of internal standards. In brief, lipids were extracted in a teflon/glass homogenizer using 2mL of methanol/chloroform (1:1, by volume) with phase separation by the addition of 1.5 mL of saline. The methanol/chloroform mixture contained the following internal standards: di-14:0 phosphatidylethanolamine (PE; 23.6 nM), di-17:0 PE (23.6 nM), di-20:0 phosphatidylcholine (PC; 11.8 nM), and di-14:0 phosphatidylserine (PS; 1.8 nM). Lipids were extracted twice from the cells and the pooled chloroform layers were washed with Folch theoretical upper phase prior to evaporation of the solvent under a nitrogen stream and re-suspended in chloroform. For fatty acid composition analysis, fatty acid methyl esters (FAMES) were prepared from total cell lipid extracts by subjecting them to strong acid hydrolysis (16.6% HCL in methanol at 75°C overnight). The FAMES were separated from other sterols by thin layer chromatography (TLC) and analyzed by gas chromatography-flame ionization detector (GC-FID). The total triglyceride content and the total cholesterol content were determined using Triglyceride Colorimetric Assay Kit (Cayman) and Cholesterol Fluorometric Assay Kit (Cayman) strictly following the manufactures protocol.

### Boyden chamber migration and invasion

Post 48 h transfection with scrambled siRNA or CBS siRNA, A2780, SKOV3 or OVCAR4 cells were serum-starved overnight, detached from culture plates and 1 × 10^5^ cells were plated into 8 μm transwell chamber in 200 μl of serum-free RPMI1640 medium. The lower chambers of the plate were supplied with 650 μl of RPMI1640 medium with 10% FBS. The cells were allowed to migrate for 12 h after which cells were fixed and stained with crystal violet solution. Cells in the upper chamber were removed using a cotton swab, and cells migrating through the membrane were counted. Cell invasion studies were performed using Boyden chamber equipped with membranes pre-coated with fibronectin (100 μg/mL; Sigma) or matrigel (1 mg/ml).

### Immunoblotting

Immunoblotting analysis was carried on cell lysates of A2780, SKOV3 or OVCAR4 in RIPA buffer supplemented with protease-phosphatase mix (Pierce). Briefly, the cell lysate was separated on 10% or 12% tris-glycine SDS-PAGE gel and transferred to PVDF membrane. Membranes were blocked in 5% non-fat dry milk in TBS with 0.1% TWEEN-20 (TBST) for 1 h at room temperature followed by incubation with indicated primary antibodies in TBST with 5% BSA. Primary Antibodies were used in dilutions recommended by the manufacturer and secondary antibodies were used at a concentration of 1:10,000. Equal loading was verified by by immunoblotting with GAPDH, actin or tubulin. Immunoblotting images were scanned and quantified with ImageJ (image processing and analysis in Java, NIH) using the loading control to normalize the experimental value.

### Real-time PCR

Total RNA was isolated from transfected cells using RNeasy Plus Mini kit (QIAGEN). RNA was first retrotranscribed using iScript cDNA Synthesis kit (Bio-Rad) and then realtime PCR was carried out using iTaq SYBR Green (Biorad) following the suppliers protocol. The primers used for qRT-PCR are listed as below: Gene symbol Forward primer sequence Reverse primer sequence

SREBP1a: 5′-CGGCGCTGCTGACCGACATC-3′ 5′-CCCTGCCCCACTCCCAGCAT-3′

SREBP1c: 5′-GCGCAGATCGCGGAGCCAT-3′ 5′-CCCTGCCCCACTCCCAGCAT-3′

SREBP2: 5′-CAAGCTTCTAAAGGGCATCG-3′ 5′-AGTAGGGAGAGAAGCCAGCC-3′

ACC1: 5′-GAGGGAAGGGAATTAGAAAA-3′ 5′-CTTGAACCTGTCTGAAGAG-3′

FASN: 5′-CACAGGGACAACCTGGAGTT-3′ 5′-ACTCCACAGGTGGGAACAAG-3′

HMGR: 5′-TGACCTTTCCAGAGCAAGC-3′ 5′-CCAACTCCAATCACAAGACATTC-3′

HMGS: 5′-CATTAGACCGCTGCTATTCT-3′ 5′-AGCCAAAATCATTCAAGGTA-3′

The comparative C_t_ method [77] was used to calculate the relative abundance of the mRNA and compared with that of 36β4.

### Confocal microscopy

Localization of SREBP was determined by immunostaining followed by confocal microscopy. Approximately, 1.5 × 10^3^ cells were plated per chamber of 4-chambered slides. After 12 h, the cells were fixed with 4% PFA, permeabilized with 0.1% TritonX-100 in PBS, blocked with 4% BSA in PBS, stained with primary SREBP antibody (dilution 1:100) or anti-Sp1 antibody (dilution 1:500) in 1% BSA-PBS, blocked with 5% goat serum in 1% BSA-PBS and stained with AlexFlour488 goat anti-rabbit secondary antibody. The cells were washed 3 × 3 min with PBS after each step during the immunostaining. The images were acquired using Olympus FV-500 microscope and processed using ImageJ (NIH).

### ChIP assay

Post 48 h of transfection, A2780 cells were treated with 18.5% formaldehyde to cross-link proteins to DNA. Chromatin immunoprecipitation (ChIP) assays were performed using the EZ-ChIP chromatin immunoprecipitation kit (Millipore; catalog no. 17–371) according to the manufacturer's instructions. Briefly, after *in vivo* cross-linking, cells were lysed and sonications were performed to shear the chromatin to a manageable size (200 to 1,000 bp). Immunoselections of cross-linked protein-DNA were performed with anti-rabbit IgG (negative control), anti-SP1 antibody, and protein G-conjugated agarose beads. Protein-DNA complexes were washed, and then protein-DNA cross-links were reversed to free DNAs. The purified DNAs were analyzed by PCR using primers for SREBP1 promoter [70]: 5′-ACTCGGCTTCCTTGCTTGGTGCTG-3′ and 5′-CGCCGGCGAAAAGTTCCTCGGA-3′.

### Preclinical model of ovarian cancer

Before injection, tumor cells were washed twice with PBS, detached by 0.1% cold EDTA, centrifuged for 7 min, and reconstituted in HBSS (Invitrogen). Cell viability was confirmed by trypan blue exclusion. For the generation of orthotopic ovarian tumor models, 50 μL of 1.5 × 10^6^ shCTL-, shCBS-, shSREBP1- or shSREBP2-A2780 cells were injected into the ovaries of nude mice (ages 6–8 wk).

### Immunohistochemistry

Tumor grafts or mouse tissues were embedded in paraffin and sectioned (4 μm). These sections were deparaffinized in xylene, rehydrated in graded alcohol, subjected to heat-induced antigen retrieval with Target Retrieval Solution, and blocked with Protein Block. Immunohistochemistry was performed according to standard protocols. Antigen retrieval was achieved by heating sections in 95°C citrate buffer for 10 minutes. Sections were incubated with specific antibodies overnight at 4°C. For CD31 (1:100) and Ki67 (1:100) staining, the dark brown signal was revealed after incubation with the ABC kit (Vector), followed by a diaminobenzidine (DAB) and hydrogen peroxide reaction using the DAB detection kit (Vector). Counterstaining was performed by incubating the slides in Hematoxylin for 5 min. Images were taken using Nikon Eclipse Ni microscope.

### Statistical analysis

All experiments were performed in triplicate and statistical analysis was performed using two-sided student's *t*-test.

## SUPPLEMENTARY FIGURES


