# Supplementary material for: Role of cystathionine beta synthase in lipid metabolism in ovarian cancer
Source: Oncotarget. 2015 Oct 6;6(35):37367–84. doi: 10.18632/oncotarget.5424 (PMC4741935; doi:10.18632/oncotarget.5424)
Supplement: Supplementary file 1 [file oncotarget-06-37367-s001.pdf]

SUPPLEMENTARY FIGURES

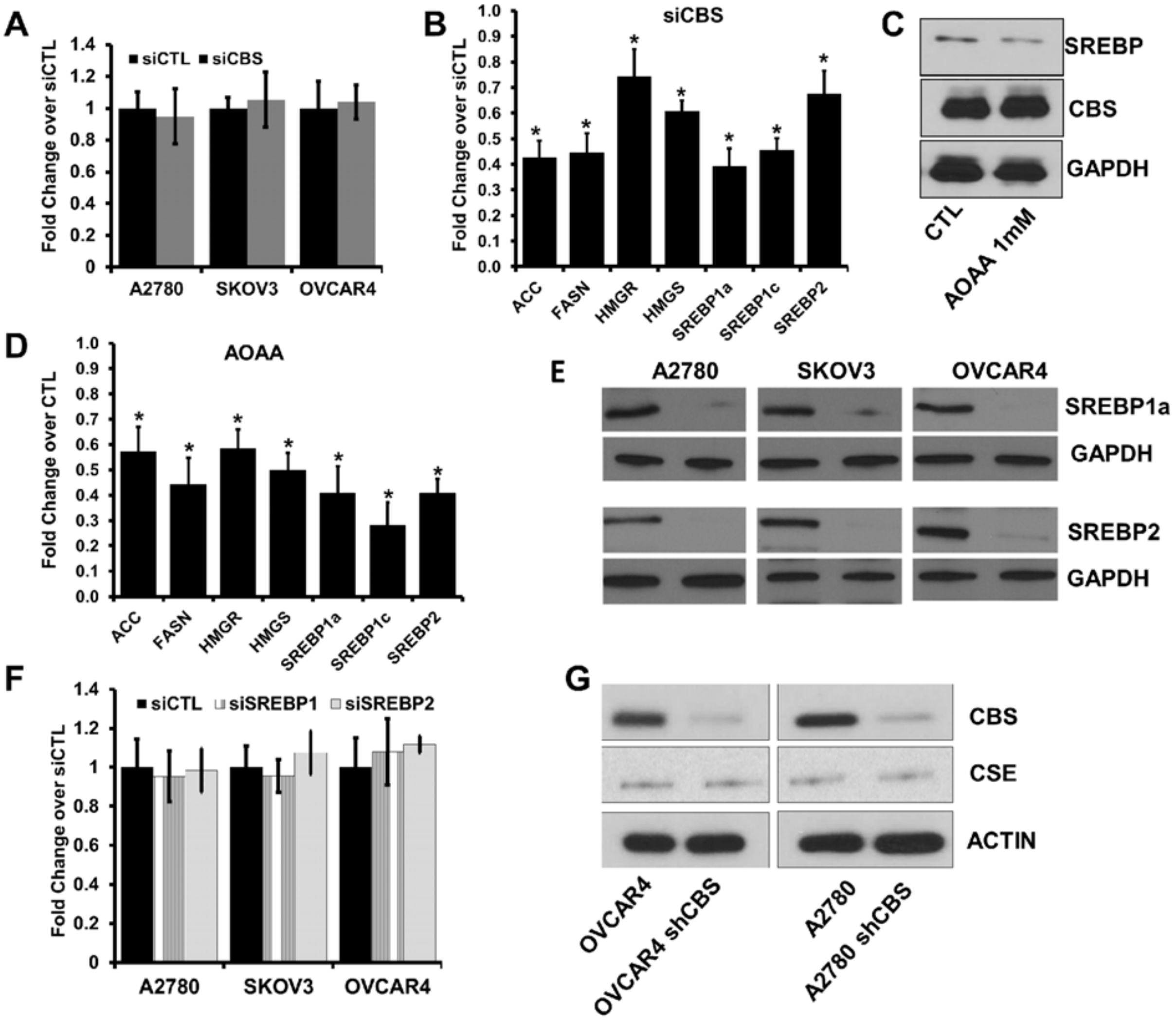

Supplementary Figure S1: A. Effect of gene silencing of CBS on A2780, SKOV3 and OVCAR4 cell proliferation at 12h. Fold change proliferation values are means ± SD. N = 3. **B.** Expression profile (qRT-PCR) of fatty acid and triglyceride biosynthesis genes in A2780 ovarian cancer cell line post CBS silencing, relative to cells with siCTL. **C.** Western blot analysis of the CBS and SREBP proteins in vehicle treated or AOAA treated cells. GAPDH is used as the loading control. **D.** Expression profile (qRT-PCR) of fatty acid and triglyceride biosynthesis genes in A2780 ovarian cancer cell line post AOAA treatment, relative to cells treated with vehicle. **E.** Western blot analysis of the SREBP1a and SREBP2 proteins in siCTL and siSREBP1 or siSREBP2 transfected cells. GAPDH is used as the loading control. **F.** Effect of gene silencing of SREBP1, SREBP2 or both on A2780, SKOV3 and OVCAR4 cell proliferation at 12h. Fold change proliferation values are means ± SD. N = 3. **G.** Expression of CBS in stable knockdown OVCAR4 shCBS and A2780 shCBS cells and effect of CBS knockdown on the expression of CSE, as determined by immunoblotting. Actin is used as a loading control.

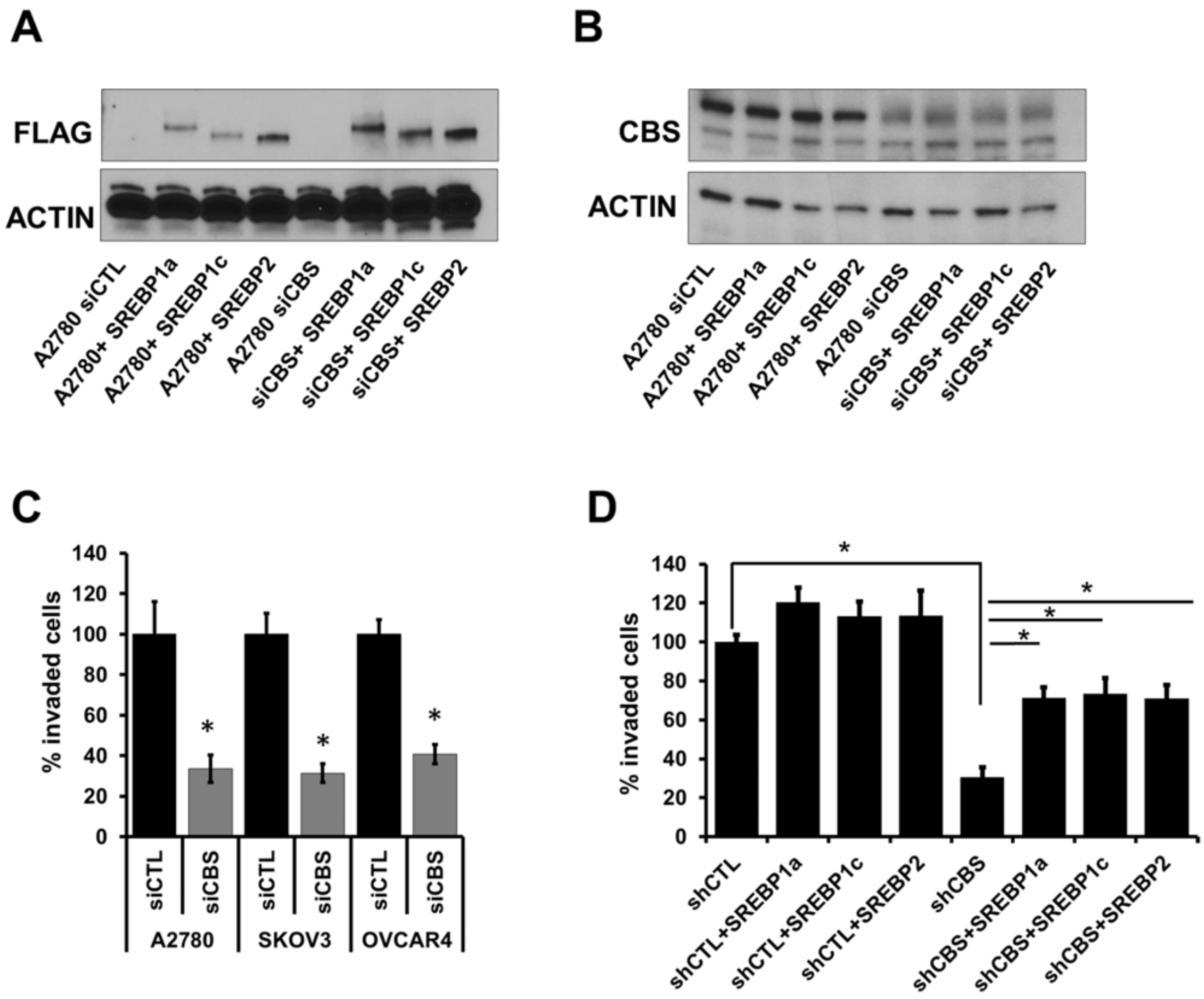

Supplementary Figure S2: A. Expression of Flag tagged SREBP in siCTL and siCBS A2780 cells. Actin is used as the loading control. B. Western blot analysis of the CBS in A2780 cells expressing Flag-SREBP. Actin is used as the loading control. **C.** Silencing of CBS inhibits cell invasion of matrigel matrix by ovarian cancer cells (A2780, SKOV3 and OVCAR4). Invasion of siCTL and siCBS cells through matrigel-coated filters was examined using Boyden chamber. Cells were fixed and stained with crystal violet and counted under microscope. Percentage change values are means ± SD. N = 3. **D.** Overexpression of SREBP1 or SREBP2 can restore invasive properties in CBS silenced cells. Transient transfection of Flag tagged -SREBP1a, -SREBP1c and -SREBP2 induces cell invasion through matrigel-coated filters in CBS knockdown A2780 cells (shCBS). Invasion of cells was examined using Boyden chamber. Cells were fixed and stained with crystal violet and counted under microscope. Percentage change values are means ± SD. N = 3. For C and D, *$P < 0.05$ versus corresponding control.
